# Oral Probiotic Expressing Human Ethanol Dehydrogenase Attenuates Damage Caused by Acute Alcohol Consumption in Mice

**DOI:** 10.1128/spectrum.04294-22

**Published:** 2023-04-11

**Authors:** Xiaoxiao Jiang, Chunlong Yan, Hanlin Zhang, Li Chen, Rui Jiang, Kexin Zheng, Wanzhu Jin, Huijuan Ma, Xiaomeng Liu, Meng Dong

**Affiliations:** a Key Laboratory of Animal Ecology and Conservation Biology, Institute of Zoology, Chinese Academy of Sciences, Beijing, China; b University of the Chinese Academy of Sciences, Beijing, China; c Agriculture College of Yanbian University, Yanji, Jilin, China; d Institute of Infectious Disease, Ditan Hospital, Capital Medical University, Beijing, China; e Department of Internal Medicine, Hebei Medical University, Shijiazhuang, Hebei, China; f Key Laboratory of Metabolic Diseases, Hebei General Hospital, Shijiazhuang, Hebei, China; g Department of Nutrition and Food Hygiene, College of Public Health, Xinxiang Medical University, Xinxiang, Henan, China; h Institute of Neuroscience and Translational Medicine, College of Life Science and Agronomy, Zhoukou Normal University, Zhoukou, China; Suranaree University of Technology

**Keywords:** human ADH1B, recombinant probiotics, alcohol decomposition, oral administration

## Abstract

Alcohol is an essential drug in human life with multiple medical functions, but excessive alcohol intake, even a single episode of binge drinking, can cause serious damage. Reducing alcohol consumption or absorption is a direct way to alleviate the related harm. Alcohol is decomposed successively by alcohol dehydrogenase (ADH) and acetaldehyde dehydrogenase (ALDH) in the liver. Here, we produced a human ADH1B (hADH1B)-expressing probiotic, a recombinant Lactococcus lactis, that aimed to enhance alcohol degradation in the intestinal tract after oral administration. Our results showed that the oral hADH1B-expressing probiotic reduced alcohol absorption, prolonged the alcohol tolerance time, and shortened the recovery time after acute alcohol challenge. More importantly, the liver and intestine were protected from acute injury caused by alcohol challenge. Therefore, the engineered probiotic has the potential to protect organ damage from alcohol consumption. Furthermore, this engineered probiotic may have beneficial effects on alcohol-related diseases such as alcoholic fatty liver disease.

**IMPORTANCE** Alcohol plays an important role in medical treatment, culture, and social interaction. However, excessive alcohol consumption or improper alcohol intake patterns can lead to serious damage to health. Aiming to reduce the harm of alcohol consumption, we designed a recombinant probiotic expressing hADH1B. Our results showed that this recombinant probiotic can reduce alcohol absorption and protect the body from alcohol damage, including hangover, liver, and intestinal damage. Reducing alcohol damage is helpful to the health of people with difficulty in abstinence. The engineered probiotic may provide new strategies for treatment and prevention of the negative effects of alcohol, and it also has the potential for widespread application.

## INTRODUCTION

Wine is an indispensable part of human civilization that serves economic, social, medical, and religious purposes. According to the latest statistics, between 1990 and 2017, per capita alcohol consumption by adults increased from 5.9 L to 6.5 L worldwide and is forecast to reach 7.6 L by 2030 (1). Large-scale population-based cohort studies have found that moderate drinking does not increase the risk of all-cause mortality among drinkers (2). However, a recent study showed that moderate drinking was associated with an increased risk of significant fibrosis progression in nonalcoholic fatty liver disease (NAFLD) (3). Binge drinking, which is characterized by the intake of large amounts of alcohol in a short time period, is a major cause of preventable impairment of health. Alcoholic beverages disturb the intestinal absorption of 79 nutrients, including several vitamins, sodium, and water (4). Alcohol consumption also affects the immune system of the gut, contributing to the immune deficiency associated with alcohol abuse (5). As alcohol enters the bloodstream, the cardiovascular system and liver are the main targets of damage, resulting in heart disease, cardiomyopathy, alcoholic fatty liver disease, hepatitis, cirrhosis, and other alcohol-associated liver diseases (6–8). Alcohol and its secondary metabolite acetaldehyde directly attack the nervous system, leading to a host of neurological problems, including anxiety, insomnia, and loss of memory (9, 10). According to the World Health Organization reported (2018), the harmful use of alcohol is a causal factor in more than 200 healthy conditions and incurs a heavy socioeconomic cost (11). Given the many different internal and external motivations and social-cognitive reasons for drinking, better compliance may be achieved by the use of antialcoholics than by abstinence from alcohol; thus, this approach may help mitigate the health problems caused by alcohol intake.

About 2 to 10% of oral alcohol is excreted through breath, urine, and sweat; the remainder is metabolized to acetaldehyde by alcohol dehydrogenase (ADH) and then quickly converted to carbon dioxide and water in a reaction catalyzed by aldehyde dehydrogenase (ALDH) in the liver (12). Large-scale phenotyping studies have consistently found that the genetic diversity of these two proteases is the main reason for the wide variation in people's tolerance to alcohol (12). A variant of ADH1B, which occurs primarily in Asian and Polynesian populations, shows higher enzymatic activity. The ALDH2*2 allele (Glu504Lys), which is also common in East Asia, is associated with adverse reactions to alcohol consumption, including facial flushing, hypotension, headaches, and nausea (13, 14). Therefore, genetic modification or external supplementation with a highly efficient enzyme may be more useful to alcoholics than drugs or foods intended to increase endogenous catalytic enzyme activity.

In theory, many gene-editing techniques, such as the CRISPR/Cas9 system, as well as adeno-associated virus and lentiviruses can be used to regulate the expression of genes. Administration of an adenoviral vector containing the human ADH1B (hADH1B) gene accelerated the breakdown of alcohol in mice (15). However, owing to safety concerns, these techniques are banned in humans. In the delivery of bioactive molecules, the efficiency of delivery and the maintenance of activity are key limiting factors. In addition, synthetic protein supplements have high requirements for enzyme purity, concentration, and half-life, but these standards are difficult to reach and the associated costs are high. Recently, bacterial engineering, which integrates genetic engineering techniques with bacterial genomics, has made it possible to create effective biomacromolecules. To date, there have been dozens of clinical studies on the use of bacteria for disease management (16). These bacteria include Escherichia coli and *Bacteroides*, which predominate in the gastrointestinal tract. The bacteria used in such studies are modified so that they do not produce virulence factors and are thus unable to induce damage on the surface of the intestinal epithelium (17, 18). Certain anaerobic bacteria, including Salmonella, can target tumors and be engineered as antitumor biological agents (19–21). Owing to the chemotaxis ability, biomolecule secretion, and other specific biological characteristics of these bacteria, their application, combined with a variety of gene-editing techniques, has greatly expanded the scope of biotherapy (22).

Lactococcus lactis, a probiotic, is a nonpathogenic, noncolonizing, food-grade bacterial strain that is commonly used in the dairy industry and has an excellent safety record. Genetically modified L. lactis can serve as a vehicle for delivery of biologically active molecules (23). In this study, the hADH1B gene was inserted into a pNZ8149 construct equipped with a GapA promoter, and L. lactis was chosen as the delivery chassis for ectopic expression of hADH1B. The results showed that our recombinant L. lactis could reduce the absorption of ethanol and improve alcohol tolerance, prolonging the alcohol tolerance time and shortening the recovery time after drinking. Further investigation showed that the intestinal inflammation and acute liver damage caused by binge drinking were both improved. These findings suggest that our recombinant probiotic has potential clinical applications as an efficacious means of alleviating alcohol-related health problems.

## RESULTS

### Preparation and characterization of oral recombinant probiotics.

**FIG 1:**
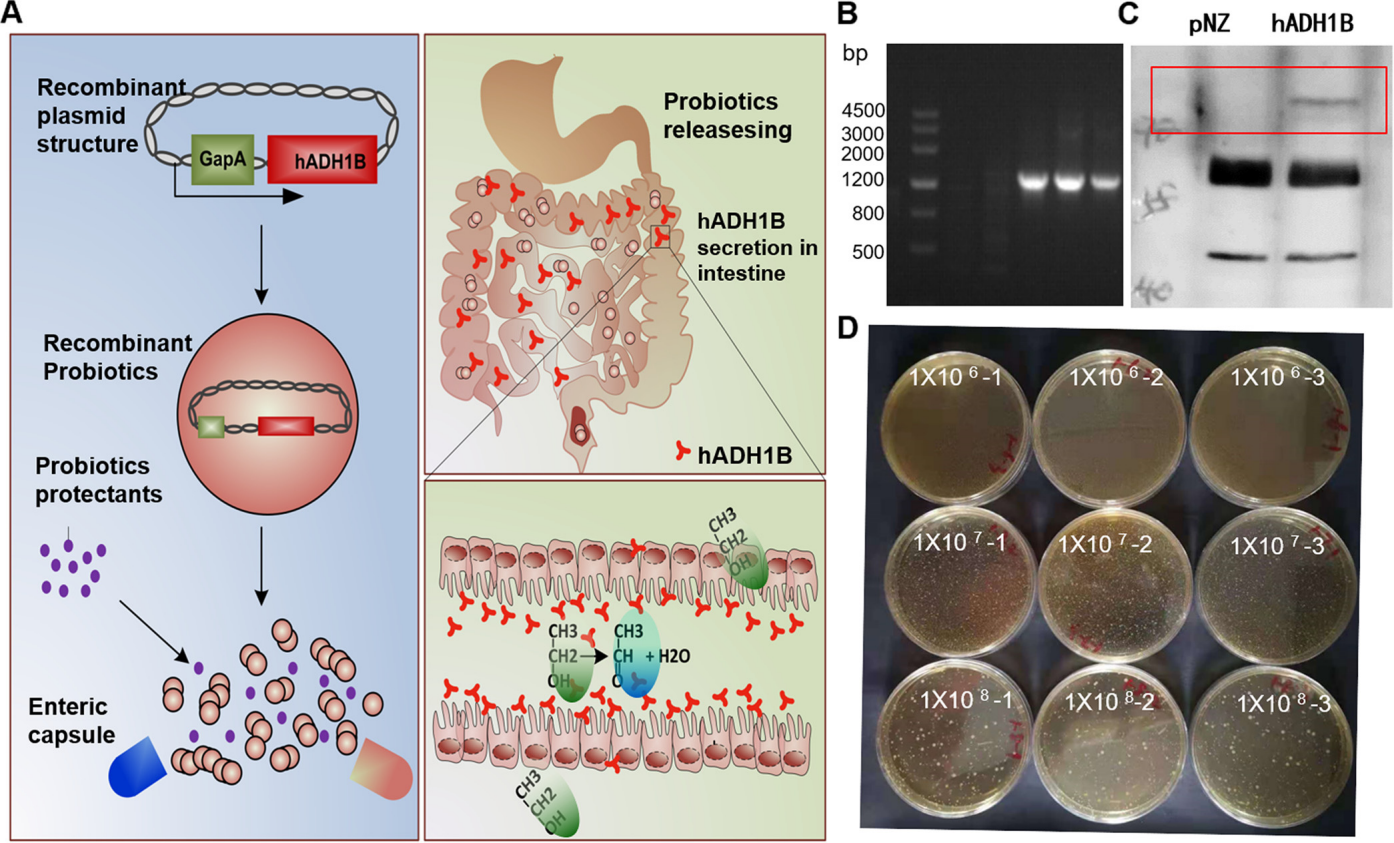
Preparation and characterization of oral recombinant probiotics for treatment of alcoholics. (A) Schematic overview of preparation and function of recombination probiotics. (B) PCR identification of hADH1B recombinant probiotics. The target DNA fragment was about 1,356 bp. (C) Immunoblots of culture supernatant to detect the secretion of hADH1B. (D) Detection of the viability of encapsulated probiotics. The final concentration of viable bacteria in the ingredients was about 4 × 10^9^ CFU mg^−1^.

pNZ8149 is a widely used probiotic expression vector, and new constructs equipped with the GapA promoter (patent no. CN111518801A) have been proved to be highly effective in driving the expression of foreign genes. To produce hADH1B-secreting recombinant probiotics, the gene was inserted into a pNZ8149-GapA construct and transferred into L. lactis by electroporation technology. An enteric capsule was used to protect the L. lactis from being killed by stomach fluids. In theory, hADH1B would be secreted in the intestine and transported into the blood (Fig. 1A). The DNA construct containing the target gene was detected by PCR (Fig. 1B) and further identified by sequencing. In our new DNA construct, the intrinsic nisin promoter was changed to the GapA promoter, followed by a secretory peptide editing sequence (Usp45), an expression enhancement element (LESS), and an enterokinase-cutting peptide editing sequence (EK). To confirm that the construct still worked as expected to produce free protease, Western blotting was conducted. The results showed that the recombinant L. lactis could specifically secrete hADH1B into the culture supernatant (Fig. 1C). Further, the hADH1B-expressing L. lactis was prepared in microcapsule particles, as this delivery method could protect bacterial activity (Fig. 1D) and could enable operation for subsequent oral experiments in mice.

### Oral recombinant probiotics prolonged alcohol tolerance time.

**FIG 2:**
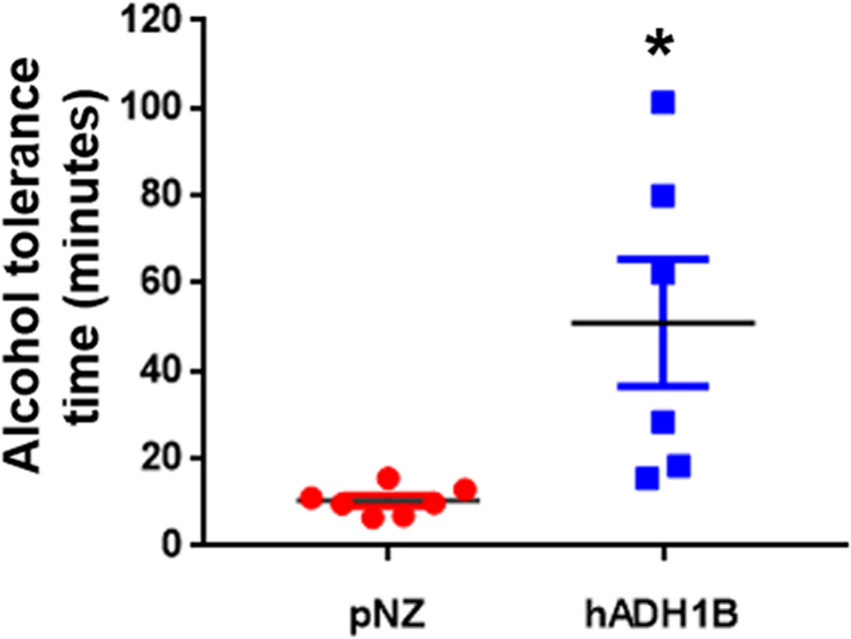
Statistical analysis of the differences in alcohol tolerance times between the two groups. hADH1B-expressing probiotics prolonged alcohol tolerance times. *, *P* < 0.05.

To develop a safe and stable model of acute alcohol challenge, a volume gradient of wine was given to mice, and 6 mg/g of body weight (BW) was chosen for the subsequent experiments, as this dosage caused loss of exercise ability in all mice within 1 h after drinking but without being life-threatening (see Table S1 in the supplemental material). We explored the intragastric dose of the recombinant probiotics in preliminary experiments. According to our results, there was no beneficial effect detected in the alcohol challenge mice when administered with 1 × 10^7^ recombinant probiotics. However, when the mice were administered recombinant probiotics at 1 × 10^8^ CFU, the alcohol tolerance time of the mice was prolonged but the effects varied greatly from batch to batch (data not shown). Other studies have also shown that a dose rate of 1 × 10^9^ CFU is safe and effective (24, 25). So, we chose the dose rate of 1 × 10^9^ CFU recombinant probiotics for the following test. One hour after 1 × 10^9^ CFU of pNZ control bacteria or hADH1B-expressing bacteria was administered by gavage, the mice were given 6 mg/g BW alcohol. The results showed that the alcohol tolerance time (i.e., the time from drinking to loss of exercise ability) was significantly prolonged in mice treated with the hADH1B-expressing probiotic (Table S2; Fig. 2). All mice in the pNZ group lost their self-righting reflex within 1,200 s, whereas nearly half of the mice in the hADH1B-expressing probiotic group were still able to move 1 h after drinking (Table S3). In conclusion, the hADH1B-expressing probiotic effectively enhanced acute alcohol tolerance and increased the alcohol intake threshold for acute intoxication.

### Oral probiotics reduced exercise recovery time in mice after alcohol challenge.

**FIG 3:**
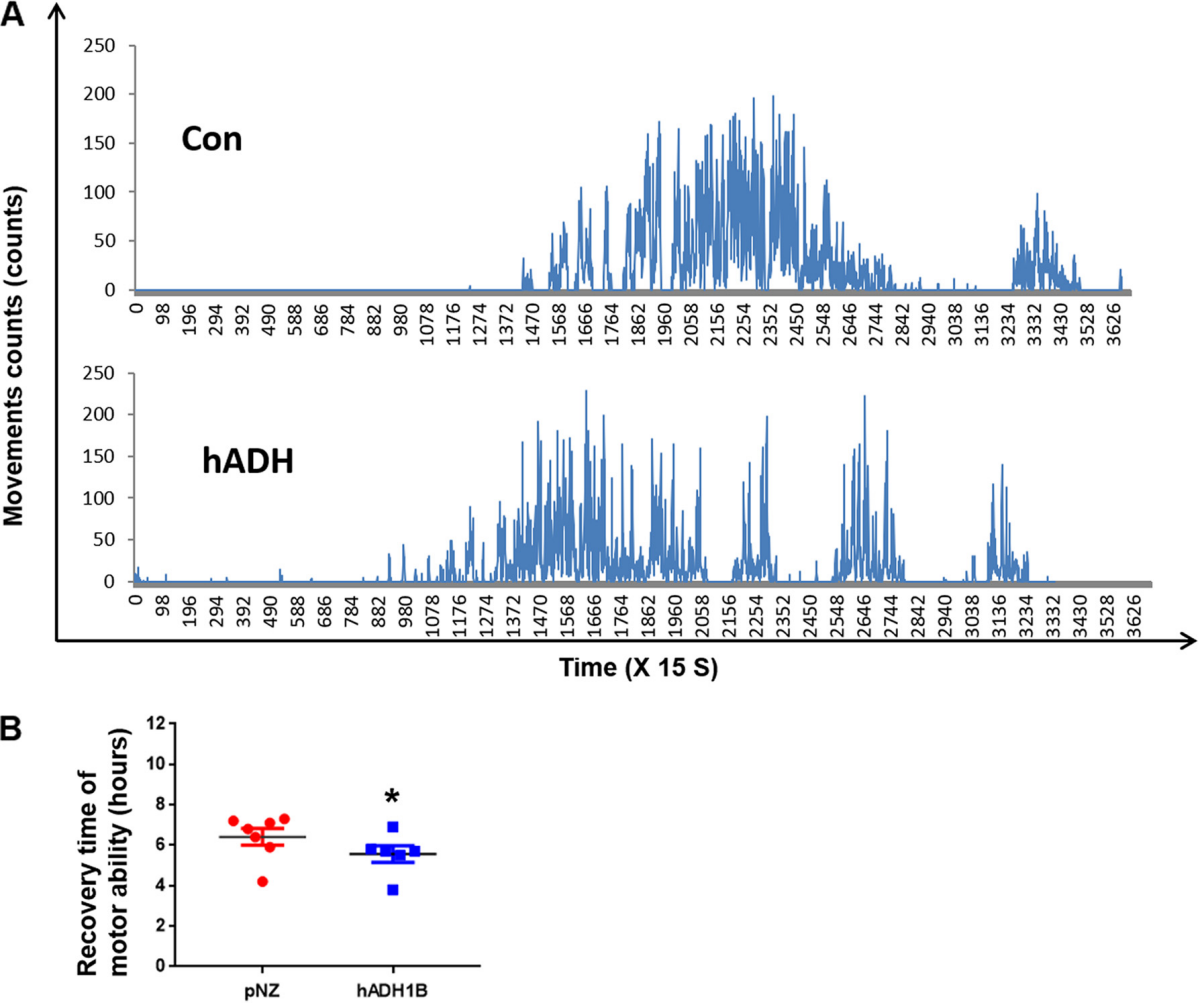
Oral probiotics reduced exercise recovery time in drunken mice. (A) Chart showing recording of recovery of movement in a drunken mouse. Con, control. (B) hADH1B recombinant probiotics shortened the motor recovery times of drunken mice. *, *P* < 0.05.

We observed that it takes 6 to 10 h for drunk mice to recover. To enable the accurate recording of the recovery times of mice in different treatment groups, the mice were placed into an exercise recorder 1 h after alcohol challenge. Exercise times were recorded every 15 s (Fig. 3A). When the recorder received nonzero data four consecutive times, the mice were considered to have recovered their locomotor ability. Statistically, we found that mice treated with hADH1B regained their exercise capacity after 5.5 ± 0.41 h (*n* = 6), significantly less than the time taken by the pNZ probiotic treatment group (6.4 ± 0.41 h, *n* = 7) (Fig. 3B). Moreover, one-quarter of the mice in the hADH1B-expressing probiotic treatment group did not lose their self-righting reflex and exercised throughout the whole process, while all the mice in the pNZ probiotic treatment lost their locomotor ability after alcohol challenge (Tables S2 and S4). These results indicate that hADH1B expression of probiotics could shorten the recovery time in mice after acute alcohol challenge.

### Oral probiotics attenuate hepatointestinal lesions associated with acute alcohol consumption.

**FIG 4:**
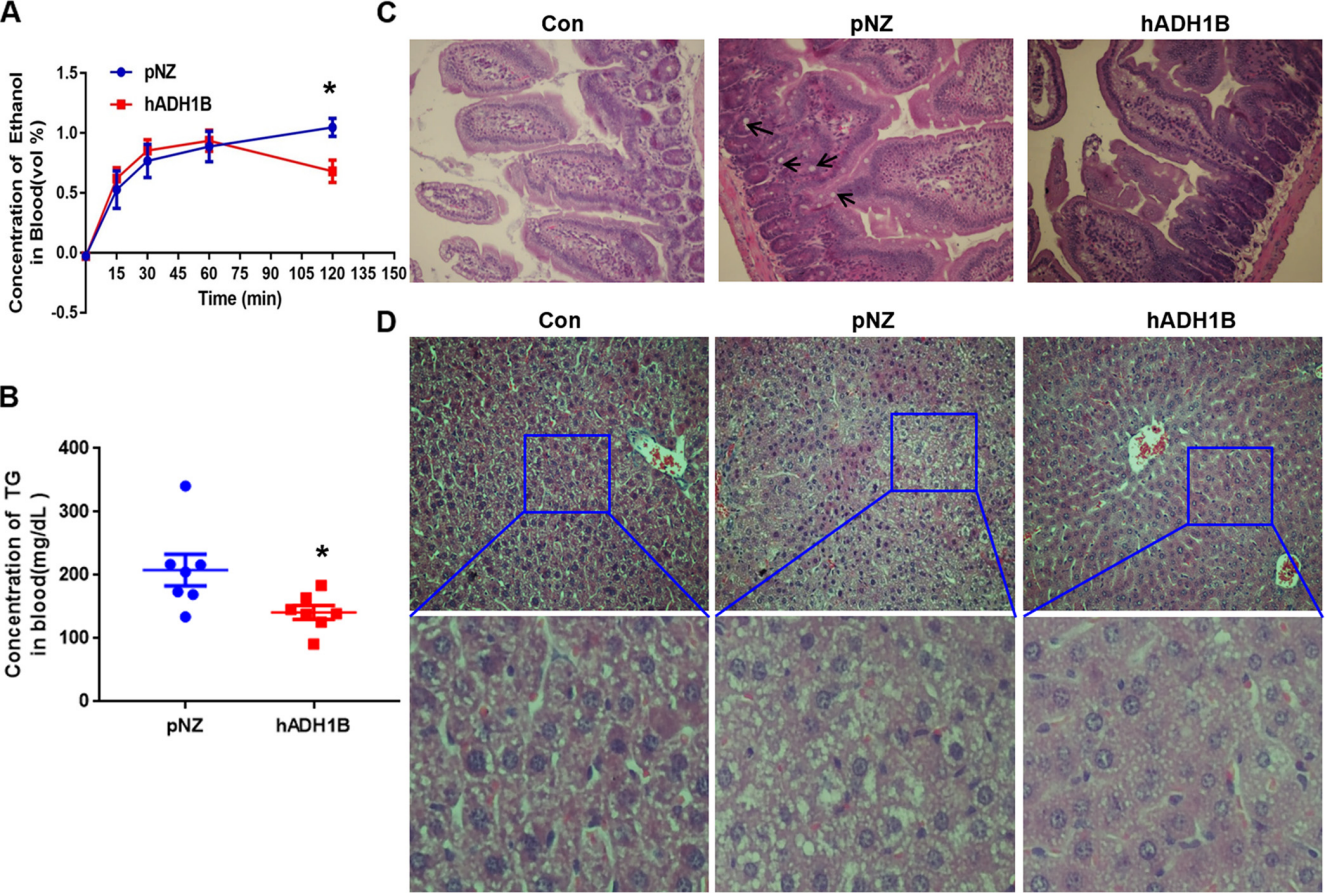
Oral probiotics attenuate hepatointestinal lesions associated with acute alcohol consumption. (A) Time course of blood alcohol residue after drinking. (B) Serum concentration of TG. (C) H&E histological staining of intestinal sections. (D) H&E histological staining of liver sections. *, *P* < 0.05.

Alcohol is absorbed mostly in the gut and eventually transported to the liver, where it is decomposed. Therefore, the gut-liver axis has an important role in regulating ethanol metabolism, and the intestine and liver are also the organs most directly damaged after drinking (5, 26). To detect acute intoxication in two groups of mice, the mucosal lesions of the intestine were observed. The goblet cells in pNZ-treated drunken mice showed much more hypertrophy than those in the nondrunken mice, and hADH1B-expressing probiotic treatment mitigated the pathogenic effects of acute alcohol consumption (Fig. 4C), indicating a reduction in alcohol absorption through the gut. Alcohol in the blood of mice reached its peak level 2 to 3 h after drinking. To test the effects of different probiotics on alcohol absorption, blood alcohol levels were measured at different points after drinking by using an EnzyChrom ethanol assay kit (BioAssay Systems; ECET-100). In the first hour, the alcohol content in the blood of mice in both groups showed no difference. Two hours after drinking, the serum alcohol residue in the pNZ group continued to increase, whereas that in the hADH group showed a significant downward trend and was significantly lower than that in the pNZ group (Fig. 4A). On further examination, we found that hADH1B treatment reduced the blood triglyceride (TG) concentrations (Fig. 4B) and, synchronously, reduced lipid levels in the livers of mice treated with hADH1B (Fig. 4D). In conclusion, treatment with hADH1B-expressing probiotics can alleviate the intestinal damage caused by acute alcohol consumption and reduce fat content in the liver and blood.

## DISCUSSION

Recently, social diversity and improvements in living standards have led to an increase in the frequency of drinking, which is likely to affect people's health (27). The immediate phenomenon after drinking is a hangover, the most common symptoms of which include tiredness, increased thirst, sleepiness, headache, dry mouth, and nausea (28). These symptoms can adversely affect an individual’s work effectiveness and daily activities, as well as having negative economic consequences (29). Alcohol intake has been shown to be associated with a variety of diseases, such as fatty liver, cirrhosis, cardiovascular and cerebrovascular diseases, and cancer (30). Therefore, the development of effective products to reduce alcohol intake is receiving increasing attention.

Approximately 90% of ethanol metabolism occurs in the liver, where ADH metabolizes ethanol to acetaldehyde and then ALDH metabolizes acetaldehyde to acetate (31). Therefore, the rapid and effective removal of excess ethanol and its metabolite acetaldehyde has an important role in preventing liver damage (32). There is evidence that individuals with the ALDH2*2 allele are highly deficient in ethanol decomposition (33). It has been suggested that Korean pear juice can stimulate ADH and ALDH to reduce alcohol levels (34), but no significant sobering effect was found in individuals with the ALDH mutant genotype (35). Moreover, alcohol metabolism can increase oxidative stress in the body (36, 37); therefore, substances with high antioxidant activity, such as Centranthera grandiflora (38) and red ginseng (39), have been proposed as candidate antialcohol products. However, other studies have found that such products do not alleviate the negative effects of alcoholism (40). At present, there are some unanswered questions about screening for effective alcohol metabolism-accelerating products. (i) What are the mechanisms by which these products activate ADH and ALDH? (ii) How effective are they in individuals with genetic mutations? (iii) How can we rapidly search for and develop products with high efficacy, high compliance, low manufacturing costs, and high efficiency of industrial production?

In the current study, we used safe food-grade probiotic L. lactis to recombine and express human ADH *in vitro* (Fig. 1). The safety and efficacy of the L. lactis oral system have been demonstrated previously (24). The gastrointestinal tract is the most heavily burdened of all tissues after alcohol consumption (41), and hypertrophy of intestinal goblet cells is induced by alcohol stimulation (42). We found that hypertrophy of intestinal goblet cells was improved in the hADH-treated group, indicating that the L. lactis oral system directly reduced the intestinal effects of alcohol (Fig. 4C). It is likely that the recombinant probiotics expressed active hADH after passing into the intestinal tract and directly decomposed alcohol, thus reducing the burden on the intestinal tract. The observed reduction of blood ethanol levels in the hADH1B treatment group also supports this view (Fig. 4A). In addition, after ethanol enters the bloodstream, lipin-1 is upregulated, thus leading to the accumulation of cytosolic lipin-1 protein, the elevation of PAP activity, and the promotion of triglyceride synthesis in the liver (43–46). Therefore, reducing the amount of alcohol in the bloodstream could prevent the accumulation of lipids in the liver. In the current study, recombinant probiotics expressing hADH1B could directly reduce the amount of ethanol entering the blood, thus achieving the effect of liver protection (Fig. 4B and D).

Studies have shown that the ADH expression level is significantly decreased in nonalcoholic steatohepatitis (47) and hepatocellular carcinoma (48). It has been also reported that active drinkers with alcohol-related cirrhosis develop cellular immune responses against ADH, and these responses are correlated with disease severity (49). In addition, high-alcohol-producing strains of Klebsiella pneumoniae have been shown to be associated with NAFLD (50). Endogenous ethanol produced by intestinal bacteria impaired mitochondrial function in NAFLD (51). These results suggest that recombinant probiotics expressing ADH1B have a great potential to combat nonalcoholic fatty liver, cirrhosis, and even hepatocellular carcinoma.

In conclusion, we found that recombinant probiotics could express hADH directly in the intestinal tract, decompose ethanol in a short time, and effectively reduce the negative effects on various organs. Most importantly, this is the first study to deliver ADH1B, which shows higher enzymatic activity, by recombinant probiotics directly, indicating the great promise of recombinant probiotics in decomposing ethanol effectively for individuals with genetic mutations of ADH and ALDH. Moreover, the recombinant probiotics are safe and easy to use and have a mature industrial production system and low production costs. The present research not only provides new strategies for treatment and prevention of the negative effects of alcohol but also paves the way for potential widespread application in the future.

## MATERIALS AND METHODS

### Cloning and expression of recombinant hADH gene.

The plasmid pNZ8149 (MoBiTec, Goettingen, Germany) equipped with a high-efficiency constitutive promoter (GapA promoter; patent no. CN: CN111518801A) was chosen as the vector structure (Fig. 1A). The human ADH1B gene (GenBank accession no. NM_000668) was cloned into multiple cloning sites, and its N terminus was fused with the Usp45-LESS-EK sequence. The expressing DNA construct was transformed into L. lactis subsp. *cremoris* NZ3900 (MoBiTec, Goettingen, Germany) by electrotransformation, and Elliker agar plates were used to screen the recombinant probiotics. The electrotransformation conditions were 2,000 V, 25 μF, and 200 Ω. Recombinant probiotics were identified by PCR using forward (5′-CATGCCATGGTCATGAAAAAAAAGATTATCTCAGCT-3′) and reverse (5′-GCTCTAGATCAAAACGTCAGGACGGTACG-3′) primers and sequenced. The recombinant probiotics were resuspended (1%) in M17 fluid medium, incubated at 30°C for 8 h, and centrifuged (4,000 × *g* for 10 min at 4°C), and the supernatant was collected. Then, proteins in the supernatant were precipitated by a low-temperature ethanol precipitation method (52). Equal amounts of protein samples were distributed in 10% sodium dodecyl sulfate polyacrylamide gels; after separation, the proteins were transferred to a polyvinylidene fluoride membrane, incubated with blocking buffer (5% fat-free milk) for 1 h at room temperature, and blotted with anti-hADH antibody (Santa Cruz, TX, USA). The membrane was then incubated with horseradish peroxidase-conjugated secondary antibodies (Beyotime, Shanghai, China) for 1 h at room temperature. Signals were visualized using a Mini Chemi 580 (Sage Creation Science Co., Beijing, China) with Super Signal West Pico chemiluminescent substrate (Pierce, Rockford, IL, USA).

### Microcapsulation of recombinant probiotics and acid resistance test.

After 6 to 8 h of culture, the recombinant probiotics were centrifuged (4,000 × *g* for 10 min at 4°C) and collected. The recombinant probiotics were suspended in 3% (wt/vol) sodium alginate solution. The suspension was dropped into soybean oil (containing 0.2% Tween 80) at a ratio of 1:5, followed by stirring for 10 min with a magnetic mixer at 600 rpm. Then, an 0.05 M CaCl_2_ solution was added slowly with stirring (600 rpm for 10 min). After centrifugation (350 × *g* for 10 min at 4°C), the microcapsules were collected, added to the lyoprotectant (19.5% maltodextrin and 2.5% skim milk powder), resuspended, transferred to a petri dish, kept at −80°C until completely frozen, and then transferred to a vacuum freeze-dryer to obtain freeze-dried recombinant probiotic microcapsules. The recombinant probiotic microcapsules were dissolved in artificial gastric acid (0.2% NaCl, pH 1.2) at stationary state for 2 h. The recombinant probiotics were released with the microcapsule breaking buffer (19:81 ratio of 0.2 M NaH_2_PO_4_ to 0.2 M Na_2_HPO_4_) at 200 rpm for 30 min at 37°C, and the effects of microcapsulation were verified by a plate count method.

### Animal studies.

All animal studies were approved by the Institutional Animal Care and Use Committee of the Institute of Zoology, Chinese Academy of Sciences. Six-week-old male C57BL/6J mice were purchased from Vital River Laboratory Animal Technology Co. and were housed in our specific-pathogen-free laboratory animal house (Institute of Zoology of Beijing, Chinese Academy of Sciences, China) at room temperature (24°C) with a 12-h light/dark cycle; for all experiments, five mice were housed in each cage and had free access to water. The diet was composed of 11.7% kcal from fat, 66.1% kcal from carbohydrates, and 22.2% kcal from protein (Medicience, Jiangxu, China).

### Detection of mouse intoxication.

Red Start (Hongxing) Erguotou (fen-flavor liquor; 56% vol.) purchased from a supermarket was chosen for use in the mouse binge-drinking model. To establish a safe and stable model of acute drunkenness, alcohol was given to the mice at different dose rates. Six-week-old male C57BL/6J mice weighing 20 to 22 g were randomly divided into three groups (*n* = 5 in each group), and then alcohol was given at 4 mg/g BW, 6 mg/g BW, and 8 mg/g BW to each of the three groups, respectively. The righting reflex was used as the criterion to determine drunkenness. Briefly, each mouse was placed on its back on the ground with its abdomen and limbs facing upward. If the mouse could not turn itself over within 30 s, it was considered to have lost its normal righting reflex (53). The time point at which the righting reflex was lost was defined as the drunkenness point, and the duration between the first drink and drunkenness was taken as the alcohol tolerance time. The alcohol tolerance times for the three groups of mice were all shorter than 20 min, so mice that did not lose their righting reflex within 1 h were considered not to show acute drunkenness. Considering the drunkenness rate and safety, a dosage of 6 mg/g BW was chosen for subsequent experiments. In the intestinal environment, the peak expression of recombinant probiotics secreting foreign proteins occurred at about 1 to 2 h (24, 54). L. lactis (1 × 10^9^ CFU) expressing hADH1B was administered to enable detection of the anti-inebriation effects of the oral recombinant probiotics (*n* = 8). L. lactis containing the pNZ construct was used as a control (*n* = 8). After 1 h, all mice were given 6 mg/g BW alcohol, and the alcohol tolerance time was recorded.

### Motor recovery monitoring.

One hour after alcohol administration, the mice were placed individually in motion detection systems to detect their movements every 15 s. The machine reads zero when no movement of mice is monitored, illustrating that the mice are drunk. When the number of movements at four consecutive time points was not zero, the mice were considered to have recovered their motor ability.

### Histological analysis.

For hematoxylin and eosin (H&E) staining, tissues fixed with 4% paraformaldehyde were embedded in paraffin, and 5-μm-thick sections were stained and observed under a 10× or 20× objective lens.

### Detection of triglyceride concentration.

Blood samples were collected into tubes containing heparin. The blood was centrifuged at 4°C for 15 min at 3,000 × *g*, and the upper plasma was extracted. The plasma triglyceride concentration was measured with a biochemistry analyzer (Cobas c 501, Roche, Sweden).

### Statistical analysis.

For the drunkenness experiment, eight mice in each group were studied. Data are expressed as the mean ± standard error of the mean. Statistically significant differences between two groups were determined using a two-tailed Student *t* test. Statistical significance was defined as a *P* of <0.05 (*, *P* < 0.05). All statistical analyses were performed using GraphPad Prism 8 software.
