# Supplementary material for: Oral Probiotic Expressing Human Ethanol Dehydrogenase Attenuates Damage Caused by Acute Alcohol Consumption in Mice
Source: Microbiol Spectr. 2023 Apr 11;11(3):e04294-22. doi: 10.1128/spectrum.04294-22 (PMC10269551; doi:10.1128/spectrum.04294-22)
Supplement: Supplemental file 1 — Tables S1 to S4. Download spectrum.04294-22-s0001.pdf, PDF file, 0.1 MB [file spectrum.04294-22-s0001.pdf]

Supplementary information

Supplementary table 1. Establishment of drunkenness model in mice; 6 mg/g BW alcohol was chosen as the treatment dose.

Alcohol dosages Via ig	Corresponding volume	Number of mice	Drunk rate	Mortality
4 mg/g BW	9.1 Ul g^{-1} BW	5	60%	0
6 mg/g BW	13.6 Ul g^{-1} BW	5	100%	0
8 mg/g BW	18.2 Ul g^{-1} BW	5	100%	60%

Supplementary table 2. Alcohol tolerance times of two groups (n = 8) of mice according to the time taken for the righting reflex to disappear.

Alcohol tolerance time (min)								
pNZ	9.7	9.6	12.9	10.9	6.4	15.5	6.9	Died
hADH1B	28.3	101	15	80	No	62.3	No	18.3

Supplementary table 3. Effects of pNZ and hADH1B on drunkenness. hADH1B recombinant probiotics reduced the drunkenness rates of mice.

	pNZ	hADH1B
Total mice	7	8
Drunk mice	7	4
Drunk rate	100%	50%

Supplementary table 4. Effects of pNZ (n = 8) and hADH1B (n = 8) on exercise recovery time. hADH1B recombinant probiotics prolonged the exercise recovery time.

Exercise recovery time (hours)								
pNZ	7.3	6.8	5.9	7.1	7.2	6.4	4.2	Died
hADH1B	5.7	5.5	5.7	3.8	No	6.9	No	5.8
